# “My future is bright…I won't die with the cause of AIDS”: ten‐year patient ART outcomes and experiences in South Africa

**DOI:** 10.1002/jia2.25184

**Published:** 2018-10-14

**Authors:** Cheryl J Hendrickson, Sophie J S Pascoe, Amy N Huber, Aneesa Moolla, Mhairi Maskew, Lawrence C Long, Matthew P Fox

**Affiliations:** ^1^ Department of Internal Medicine School of Clinical Medicine Faculty of Health Sciences Health Economics and Epidemiology Research Office University of the Witwatersrand Johannesburg South Africa; ^2^ Department of Global Health Boston University School of Public Health Boston MA USA; ^3^ Department of Epidemiology Boston University School of Public Health Boston MA USA

**Keywords:** South Africa, HIV/AIDS, long‐term ART treatment outcomes, loss to follow‐up, attrition, adherence

## Abstract

**Introduction:**

South Africa is moving into a new era of HIV treatment with “treat all” policies where people may be on treatment for most of their lives. We need to understand treatment outcomes and facilitators of long‐term antiretroviral treatment (ART) adherence and retention‐in‐care in the South African context. In one of the first studies to investigate long‐term treatment outcomes in South Africa, we aimed to describe ten‐year patient outcomes at a large public‐sector HIV clinic in Johannesburg and explore patient experiences of the treatment programme over this time in order to ascertain factors that may aid or hinder long‐term adherence and retention.

**Methods:**

We conducted a cohort analysis (n = 6644) and in‐depth interviews (n = 24) among HIV‐positive adults initiating first‐line ART between April 2004 and March 2007. Using clinical records, we ascertained twelve‐month and ten‐year all‐cause mortality and loss to follow‐up (LTF). Cox proportional hazards regression was used to identify baseline predictors of attrition (mortality and LTF (>3 months late for the last scheduled visit)) at twelve months and ten years. Twenty‐four patients were purposively selected and interviewed to explore treatment programme experiences over ten years on ART.

**Results:**

Excluding transfers, 79.5% (95% confidence intervals (CI): 78.5 to 80.5) of the cohort were alive, in care at twelve months dropping to 35.1% (95% CI: 33.7 to 36.4) at ten years. Over 44% of deaths occurred within 12 months. Ten‐year all‐cause mortality increased, while LTF decreased slightly, with age. Year and age at ART initiation, sex, nationality, baseline CD4 count, anaemia, body mass index and initiating regimen were predictors of ten‐year attrition. Among patients interviewed, the pretreatment clinic environment, feelings of gratitude and good fortune, support networks, and self‐efficacy were facilitators of care; side effects, travel and worsening clinical conditions were barriers. Participants were generally optimistic about their futures and were committed to continued care.

**Conclusions:**

This study demonstrates the complexities of long‐term chronic HIV treatment with declining all‐cause mortality and increasing LTF over ten years. Barriers to long‐term retention still present a significant challenge. As more people become eligible for ART in South Africa under “treatment for all,” new healthcare delivery challenges will arise; interventions are needed to ensure long‐term programme successes continue.

## 1 Introduction

The 2015 World Health Organization (WHO) recommendation to offer treatment to all HIV‐positive persons regardless of CD4 count 1 has compressed the HIV care cascade, removing several key barriers to initiation and making many more patients eligible for treatment 2, 3, 4. While intervening and monitoring the early stages of HIV care is critical to achieving UNAIDS 90‐90‐90 targets 5, the long‐term success of national antiretroviral treatment (ART) programmes is also dependent on long‐term retention and viral suppression.

South Africa, which began scale‐up of its HIV treatment programme in 2004, continues to bear the brunt of the HIV epidemic with over seven million people currently living with HIV and an adult (15 to 49 years) prevalence of 19.2% (18.4% to 20.0%) in 2015 6. Over the 14 years since the ART programme scale‐up began, the country has experienced a dramatic increase in patients on ART, with an estimated 3.4 million people on treatment in 2016 7, 8. Prior to programme rollout, adult life expectancy was 49.2 years; by 2011, this had increased to 60.5 years, a huge reduction in mortality and the direct result of the successful implementation of the national treatment programme 9, 10.

Substantial research has investigated the short‐ and medium‐term clinical outcomes of people on ART and these studies demonstrate favourable treatment outcomes for cohorts in low‐ and middle‐income countries (LMICs) 11, 12, 13, 14, 15, 16, 17, 18, 19, 20, 21, 22. More recently, several studies in low‐resource settings have published longer term outcomes 23, 24, 25, 26, 27; however, in South Africa, apart from two longer term studies 17, 28, little is yet known about treatment outcomes beyond the first five years on ART in this context and retention‐in‐care remains suboptimal 29. Even less is known about the experiences and perceptions of people who have been on treatment since the start of the programme and how these may have changed as the dramatic effects of ART became commonplace. The next ten years of treatment in South Africa are going to look very different, not least with respect to ART availability and changes in eligibility criteria with the implementation of “treatment for all.” South Africa is entering an era where people may be on treatment for most of their lives, making it crucial to learn long‐term ART patients who have overcome substantial barriers to be retained in care. In order to better prepare for this next era, we sought to understand the valuable experiences of these “success stories,” patients who had successfully navigated ten years on ART, by understanding the barriers they faced in the early days of treatment as well as the factors that have contributed, and continue to contribute, to their successful decade of treatment. To further understand their experience, we put this in the context of treatment outcomes for a full cohort of patients who started ART in the first few years of large‐scale access to public‐sector HIV treatment. This study contributes to the expanding, but as yet limited, body of work on long‐term outcomes on ART in South Africa; this is important as the growing cohort of people on ART begin to navigate this next stage in their care.

## 2 Methods

This mixed methods study comprised a quantitative cohort analysis of prospectively collected data and a qualitative study among HIV‐positive patients attending a public‐sector HIV clinic in South Africa, since the early years of the national treatment programme (2004 to 2007).

### 2.1 Study site, population and data collection

We conducted this study at a large public‐sector HIV clinic in Johannesburg, Gauteng Province, South Africa. Gauteng had an estimated HIV prevalence of 12.4% in 2012 and has the second largest number of patients on ART in South Africa 8. The clinic is located in an ambulatory care wing of a large urban public‐sector teaching hospital. It is one of the largest comprehensive care, management and treatment sites in South Africa, receives PEPFAR support through a South African nongovernmental organization, and provides HIV testing, pre‐ART care and ART 30, 31. The clinic, opened in 2000, was one of the first to provide ART in the public sector, and since 2004, has initiated more than 30,000 people on treatment and provided HIV care for over 40,000. The clinic has always followed the South African Department of Health (DoH) National ART guidelines, the first of which stipulated that non‐pregnant adults with a CD4 count of <200 cells/mm^3^ or WHO Stage 4 condition were eligible for ART 32. These guidelines are relevant to this study though South Africa has since moved through other guidelines to the current “treat all” approach 33, 34. At this clinic, if a patient misses a scheduled appointment, counsellors attempt to contact them and return them to care or ascertain their vital status. Mortality is routinely determined through family or hospital report, active tracing and linkage with the South African National Vital Registration System (VRS) which allows for correction of outcomes (loss to follow‐up (LTF) to death) 30.

For the quantitative aspect, we used a cohort study design to analyse routinely collected data from this clinic. Our study population included ART‐naïve, HIV‐positive adults (≥18 years old) initiated on a standard first‐line ART regimen between 1/4/2004 and 31/3/2007 at the clinic 32. Anonymized routine data were collected prospectively at clinic visits in an electronic medical record (TherapyEdge‐HIV^TM^). Patient information collected at ART initiation and subsequent visits included demographics, laboratory and clinical data.

For the qualitative component, eligible patients were identified and recruited purposively through medical record review, while trying to ensure that age and sex were balanced and representative of the larger cohort attending the clinic at that time. Patients in the cohort were eligible if they were ≥18 years old at the time of study recruitment, initiated ART between April 2004 and March 2005, were still on treatment and active in care at the clinic at the time of enrolment and were comfortable speaking English. We were able to determine eligible patients’ next scheduled visits through the electronic medical records and approached them at the clinic on this day to assess interest in taking part in the study. Recruitment took place during routine clinic visits between October 2015 and March 2016; 32 patients were approached, of whom 24 were eligible and enrolled resulting in a response rate of 75%. We obtained written informed consent from all those interviewed and conducted interviews using a guide that incorporated themes around experiences at HIV testing, ART initiation and continued adherence. Interviews were conducted in English by a researcher not affiliated with the clinic and digitally recorded with participants’ consent.

### 2.2 Outcomes

The outcomes for this study were assessed at twelve months and ten years following ART initiation and included: (1) all‐cause mortality; (2) LTF (defined as ≥3 months late for the next scheduled appointment at this clinic); (3) attrition (defined as either death or LTF); and (4) formal transfer (defined as a documented intention to transfer treatment to another clinic). All‐cause mortality data were collected through passive reporting at the clinic along with matching to the VRS in April 2016. Linking with this registry is done by matching an individual's unique South African identification number in the patient electronic database with the deaths in the VRS; only those with a legitimate ID can be matched. The VRS is estimated to be about 94% complete for adult deaths 35. Prior research on patients at this clinic indicated that after matching to the VRS, all‐cause mortality more than doubled from 4.2% to 10.0% 36. The dataset was closed in April 2017 to allow at least ten years of follow‐up for all patients. Person‐time for 12‐month outcomes started at ART initiation and ended at the earliest date of death, LTF, transfer, completion of 12 months of follow‐up or dataset closure on 31 March 2008. Person‐time for the ten‐year outcomes started at ART initiation and ended at the earliest date of death, LTF, transfer, completion of ten years of follow‐up or dataset closure on 10 April 2017.

### 2.3 Statistical analyses

Data were cleaned, coded and analysed using SAS v9.4 (SAS Institute Inc., Cary, NC). Baseline demographic, clinical and immunological characteristics at ART initiation were described using proportions, medians and interquartile ranges (IQRs) by year that treatment was started (ART initiated 1/Apr/2004 to 31/Mar/2005 (Y1 cohort); 1/Apr/2005 to 31/Mar/2006 (Y2 cohort); and 1/Apr/2006 to 31/Mar/2007 (Y3 cohort)) and are disaggregated by sex. We also compared rates of death and LTF at twelve months and ten years by age at ART initiation and sex, and present rates per 100 person‐years with 95% confidence intervals (CIs).

Due to the variation in time to attrition, Cox proportional hazards models were used to evaluate the relationships between baseline characteristics and attrition at twelve months and ten years after ART initiation; hazard ratios (HRs) with 95% CIs are presented 37. Crude Kaplan–Meier curves showing ten‐year attrition by year of ART initiation, sex, age and baseline CD4 counts are shown and log‐rank tests were done. We evaluated the proportional hazard assumption using “log‐log” plots; the plot lines were approximately parallel, indicating that the assumption was not violated. All baseline characteristics which resulted in a non‐null effect in crude analyses were included in the respective adjusted models. Additionally, potential confounders known through prior knowledge or hypothesized to be associated with attrition were included in the adjusted models. Demographic and clinical characteristics considered *a priori* included age, sex, CD4 cell count and WHO stage.

Qualitative interviews were transcribed verbatim, checked for accuracy and completeness and entered into NVivo for thematic analysis (QSR International's NVivo 10 qualitative data analysis Software). Initially, deductive, pre‐assigned codes were used based on a conceptual framework built around decision‐making and experiences at different stages of the care continuum, followed by inductive code development and clustering into higher level categories to deepen the analysis, contextualize the findings and develop conceptualizations about them (Table S1).

### 2.4 Ethical approval

This study was approved by the Human Research Ethics Committee of the University of Witwatersrand (M150413). The analysis of de‐identified data was approved by the Boston University IRB.

## 3 Results

**Figure 1:**
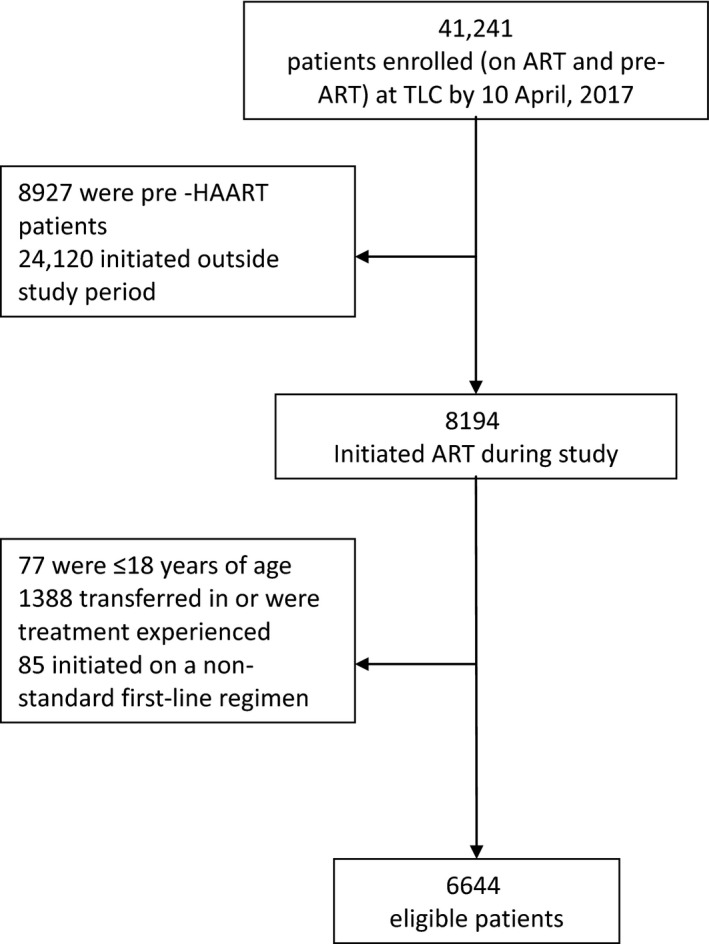
Cohort profile of patients at an HIV Clinic in Johannesburg, South Africa.

In total, 6644 patients were included in the cohort analysis; 4677 (70.4%) had valid South African IDs and were linked to the VRS (Figure 1). The number of patients initiated on ART increased each year, from 1824 patients in year 1 (Y1) to 2631 in year 3 (Y3) (Table 1). The proportion of men increased from 30.8% (Y1) to 36.1% (Y3). There was little variation in the median age at initiation, baseline CD4 counts and body mass index (BMI). The prevalence of TB at ART initiation varied slightly with 13.8% of Y1, 21.4% of year 2 (Y2) and 17.4% of Y3 patients with TB. Consistent with recommendations at the time, almost all patients were initiated on stavudine, lamivudine and efavirenz.

**Table 1:** Baseline characteristics, twelve‐month and ten‐year outcomes of patients on ART for ten years at an HIV clinic in Johannesburg, South Africa (n = 6644)

** **	Year 1 (n = 1824)^a^	Year 2 (n = 2189)^a^	Year 3 (n = 2631)^a^	Total (N = 6644)
	N (%)	N (%)	N (%)	N (%)
Total N	1824 (27.5)	2189 (32.9)	2631 (39.6)	6644
*Patient baseline characteristics*				
Sex				
Male	562 (30.8)	718 (32.8)	950 (36.1)	2230 (33.6)
Female	1262 (69.2)	1471 (67.2)	1681 (63.9)	4414 (66.4)
Age at HAART initiation (years)				
Median (IQR)	35.3 (30.4 to 41.7)	34.5 (30.1 to 40.4)	35.6 (30.7 to 42.1)	35.2 (30.4 to 41.1)
Males	37.9 (32.8 to 43.8)	36.8 (32.7 to 42.7)	37.5 (32.8 to 43.4)	37.2 (32.8 to 43.3)
Females	34.3 (29.6 to 40.5)	33.1 (29.1 to 39.1)	34.4 (29.2 to 40.9)	34.0 (29.3 to 40.1)
18 to 24.9	84 (4.6)	154 (7.0)	153 (5.8)	391 (5.9)
25 to 29.9	321 (17.6)	378 (17.3)	421 (16.0)	1120 (16.9)
30 to 39.9	865 (47.4)	1082 (49.4)	1217 (46.3)	3164 (47.6)
40 to 49.9	402 (22.0)	427 (19.5)	635 (24.1)	1464 (22.0)
>50	152 (8.3)	148 (6.8)	205 (7.8)	505 (7.6)
Nationality				
Non‐South African	44 (2.4)	98 (4.5)	137 (5.2)	279 (4.2)
South African	1780 (97.6)	2090 (95.5)	2493 (94.8)	6363 (95.8)
Males	545 (97.0)	685 (95.4)	883 (93.0)	2113 (94.8)
Females	1235 (97.9)	1405 (95.5)	1610 (95.8	4250 (96.3)
Missing	0 (0.0)	1 (0.0)	1 (0.0)	2 (0.0)
CD4 count category (cells/mm^3^)				
Median (IQR)	89 (39 to 152)	92 (32 to 163)	89 (29 to 164)	90 (33 to 161)
Males	83 (35 to 142)	62 (19 to 140)	74 (22 to 153)	74 (24 to 145)
Females	92 (41 to 155)	106 (43 to 172)	97 (35 to 169)	98 (39 to 167)
0 to 100	1008 (55.3)	1153 (52.7)	1423 (54.1)	3584 (53.9)
101 to 200	619 (33.9)	778 (35.5)	833 (31.7)	2230 (33.6)
201 to 350	156 (8.6)	212 (9.7)	299 (11.4)	667 (10.0)
351 to 500	32 (1.8)	26 (1.2)	46 (1.7)	104 (1.6)
>500	5 (0.3)	12 (0.5)	16 (0.6)	33 (0.5)
Missing	4 (0.2)	8 (0.4)	14 (0.5)	26 (0.4)
WHO Stage				
Stage 1/2	1033 (56.6)	1120 (51.2)	1347 (51.2)	3500 (52.7)
Males	281 (50.0)	293 (40.8)	440 (46.3)	1014 (45.5)
Females	752 (59.6)	827 (56.2)	907 (54.0)	2486 (56.3)
Stage 3/4	791 (43.4)	1069 (48.8)	1284 (48.8)	3144 (47.3)
Males	281 (50.0)	425 (59.2)	510 (53.7)	1216 (54.5)
Females	510 (40.4)	644 (43.8)	774 (46.0)	1928 (43.7)
Anaemia^b^				
Median (IQR)	11.5 (10.0 to 12.9)	11.7 (10.3 to 13.0)	11.3 (9.8 to 12.8)	11.5 (9.8 to 13.0)
Males	12.6 (11.0 to 14.1)	11.8 (10.1 to 13.9)	12.3 (10.6 to 13.9)	12.2 (10.5 to 13.9)
Females	11.4 (10.0 to 12.6)	11.1 (9.8 to 12.4)	11.1 (9.5 to 12.5)	11.2 (9.7 to 12.5)
None	730 (40.0)	731 (33.4)	932 (35.4)	2393 (36.0)
Mild	438 (24.0)	465 (21.2)	592 (22.5)	1495 (22.5)
Moderate	556 (30.5)	801 (36.6)	901 (34.2)	2258 (34.0)
Severe	91 (5.0)	148 (6.8)	180 (6.8)	419 (6.3)
Missing	9 (0.5)	44 (2.0)	26 (1.0)	79 (1.2)
BMI^c^				
Median (IQR)	21.9 (19.4 to 25.3)	21.5 (19.0 to 24.8)	21.6 (19.0 to 24.7)	21.7 (19.1 to 25.0)
Males	20.5 (18.6 to 22.7)	19.9 (18.1 to 22.0)	20.2 (18.5 to 22.4)	20.2 (18.4 to 22.4)
Females	22.7 (19.9 to 26.2)	22.8 (19.8 to 26.3)	22.7 (19.6 to 26.2)	22.8 (19.7 to 26.2)
Underweight	295 (16.2)	428 (19.6)	485 (18.4)	1208 (18.2)
Normal	929 (50.9)	1116 (51.0)	1376 (52.3)	3421 (51.2)
Overweight	307 (16.8)	350 (16.0)	414 (15.7)	1071 (16.1)
Obese	148 (8.1)	158 (7.2)	167 (6.3)	473 (7.1)
Missing	145 (7.9)	137 (6.3)	189 (7.2)	471 (7.1)
TB	251 (13.8)	469 (21.4)	457 (17.4)	1177 (17.7)
Males	100 (17.8)	208 (29.0)	196 (20.6)	504 (22.6)
Females	151 (12.0)	261 (17.7)	261 (15.5)	673 (15.3)
Baseline HAART regimen^d^				
NRTI				
Stavudine	1797 (98.5)	2117 (96.7)	2546 (96.8)	6460 (97.2)
Zidovudine	27 (1.5)	72 (3.3)	85 (3.2)	184 (2.8)
NNRTI or PI				
Efavirenz	1558 (85.4)	1769 (80.8)	2221 (84.4)	5548 (83.5)
Nevirapine	169 (9.3)	130 (5.9)	206 (7.8)	505 (7.6)
Lopinavir‐ritonavir	97 (5.3)	290 (13.2)	204 (7.8)	591 (8.9)
*Outcomes on HAART*				
12‐month outcomes				
Total person‐years	1650	1918	2278	5846
Outcome^e^				
Alive	1511 (84.9)	1707 (78.7)	1991 (76.5)	5209 (79.5)
Dead	129 (7.2)	180 (8.3)	233 (9.0)	542 (8.3)
Lost to follow‐up	140 (7.9)	281 (13.0)	379 (14.6)	800 (12.2)
Transferred	44	21	28	93
Attrition rate per 100 person‐years including transfers (95% CI)	16.3 (14.5 to 18.4)	24.0 (21.9 to 26.3)	26.9 (24.8 to 29.1)	23.0 (21.8 to 24.2)
Attrition rate per 100 person‐years excluding transfers (95% CI)	16.5 (14.6 to 18.6)	24.1 (22.0 to 26.4)	27.1 (25.0 to 29.3)	23.1 (21.9 to 24.4)
Median days to attrition (IQR)	131 (112 to 233)	137 (122 to 214)	132 (122 to 230)	134 (122 to 224)
Had a twelve‐month viral load reported	1198 (65.7)	1371 (62.6)	1672 (63.5)	4241 (63.8)
10‐year outcomes				
Total person‐years (median; IQR)	9826 (4.7; 2.1 to 10.0)	10,248 (3.4; 1.4 to 9.6)	11,627 (2.9; 1.1 to 9.7)	31,700 (3.7; 1.4 to 10.0)
Outcome^e^				
Alive	509 (39.5)	537 (33.3)	648 (33.5)	1694 (35.1)
Dead	344 (26.7)	427 (26.5)	456 (23.6)	1227 (25.4)
Lost to follow‐up	434 (33.7)	647 (40.2)	831 (42.9)	1912 (39.6)
Transferred	537	578	696	1811
Attrition rate per 100 person‐years including transfers (95% CI)	7.9 (7.4 to 8.5)	10.5 (9.9 to 11.1)	11.1 (10.5 to 11.7)	9.9 (9.6 to 10.3)
Attrition rate per 100 person‐years excluding transfers (95% CI)	10.4 (9.7 to 11.1)	13.4 (12.6 to 14.2)	13.9 (13.1 to 14.7)	12.7 (12.2 to 13.1)
Median months to attrition (IQR)	24.3 (7.2 to 61.7)	17.2 (5.0 to 44.2)	13.3 (4.7 to 40.8)	17.2 (5.0 to 46.2)

IQR, interquartile range; BMI, body mass index; WHO, World Health Organization; ART, Antiretroviral therapy; Hb, haemoglobin; CI, confidence interval; PI, protease inhibitor.

a Year 1: ART initiated between 1 April 2004 and 31 March 2005; Year 2: ART initiated between 1 April 2005 and 31 March 2006; Year 3: ART initiated between 1 April 2006 and 31 March 2017. Baseline CD4, haemoglobin and BMI were defined as the closest value to the date of ART initiation around a window of 12 months prior to 14 days after.

b Anaemia is defined as: none if haemoglobin ≥12 g/dL for females and ≥13 g/dL for males; mild if haemoglobin 11.0 to 11.9 g/dL for females and 11.0 to 12.9 g/dL for males; moderate if haemoglobin between 8.0 and 10.9 g/dL for females and males; and severe if haemoglobin ˂8.0 g/dL for females and males (according to WHO guidelines).

c BMI categories are defined as: underweight if calculated BMI is <18.5 kg/m^2^; normal if BMI is between 18.5 and 24.9 kg/m^2^; overweight if BMI is between 25.0 and 29.9 kg/m^2^ and obese if BMI is ≥30.0 kg/m^2^.

d Only standard 2004 ART guideline drugs included.

e Denominator excludes those who formally transferred out of care.

### 3.1 Twelve‐month and ten‐year outcomes

In the first 12 months on ART, patients contributed 5846 person‐years, including those who formally transferred out of care (n = 93, of whom 10 (10.8%) were down‐referred). Mortality and 12‐month LTF increased each year (7.1%, 8.2% and 8.9% of Y1, Y2 and Y3 patients respectively died, and 7.7%, 12.8% and 14.4% of Y1, Y2 and Y3 patients respectively were lost to follow‐up by 12 months). The resulting attrition rates (death or LTF) were 16.3, 24.0 and 26.9/100 person‐years in Y1, Y2 and Y3 respectively. Excluding those who were transferred for care to another clinic, we see a similar pattern at twelve months with increases in both mortality and LTF for each of the first three years with little change in the proportion who died or were lost to follow‐up (Table 1).

When looking at ten‐year outcomes, including transfers, patients contributed 31,700 person‐years, with a median follow‐up time of 3.7 (IQR: 1.4 to 10.0) years. After ten years of follow‐up, 27.9% of Y1 patients were still alive and in care, while 18.9% had died, 23.8% were lost to follow‐up and 29.4% transferred. Mortality by ten years remained relatively similar for Y1, Y2 and Y3; however, the proportion lost to follow‐up increased from 23.8% among Y1 patients to 29.6% in Y2 and 31.6% in Y3 patients. These resulted in increasing rates of attrition between these cohorts from 7.9/100 person‐years in Y1 to 11.1/100 person‐years in Y3. Excluding the 27.3% who were transferred, the proportion still alive in care increased to 39.5% of Y1 patients, 33.3% of Y2 patients and 33.5% of Y3 patients at ten years. The proportion of those who died decreased from 26.7% among Y1 to 23.6% among Y3 patients while those lost to follow‐up increased from 33.7% among Y1 to 42.9% among Y3. The rates of attrition increased when transfers were excluded, rising from 10.4/100 person‐years among Y1 to 13.9/100 person‐years in Y3. The median months to attrition decreased between these cohorts as well, from 24.3 (IQR: 7.2 to 61.7) in Y1 to 13.3 (IQR: 4.7 to 40.8) among Y3. Of those who were transferred (n = 1811), 58.0% (n = 1050) had been down‐referred.

We also assessed rates of death and LTF by age at ART initiation and sex at ten years (Table 2). Over this time, the all‐cause mortality rates showed little difference by age at initiation with the exception of those aged 50 and older whose ten‐year mortality rates were 6.6/100 person‐years compared to 3.9/100 person‐years among all patients. Ten‐year LTF rates varied little by sex overall but were higher among those who initiated at a younger age. Again the rates between males and females varied little and the rates decreased with age from 9.7/100 person‐years among those aged 18 to 24 years at ART initiation to 5.2/100 person‐years among those aged 50 years and older.

**Table 2:** Cumulative rates of death and loss to follow‐up at ten years by gender and age among patients who initiated ART between April 2004 and March 2007

Outcome	Age at initiation	Female (n/N (%))	Rate per 100 person‐years (95% CI)	Male (n/N (%))	Rate per 100 person‐years (95% CI)	Overall rate per 100 person‐years (95% CI)	Relative Rate per 100 person‐years (95% CI)^a^
Deaths	Overall	712/4414 (16.1)	3.3 (3.1 to 3.6)	515/2230 (23.1)	5.0 (4.6 to 5.4)	3.9 (3.7 to 4.1)	1.49 (1.32 to 1.67)
	18 to 24.9	50/349 (14.3)	3.3 (2.5 to 4.4)	8/42 (19.1)	3.8 (1.9 to 7.7)	3.4 (2.6 to 4.4)	1.15 (0.47 to 2.44)
	25 to 29.9	124/896 (13.8)	3.1 (2.6 to 3.6)	38/224 (17.0)	3.8 (2.7 to 5.2)	3.2 (2.7 to 3.7)	1.23 (0.83 to 1.79)
	30 to 39.9	325/2049 (15.9)	3.2 (2.9 to 3.6)	236/1115 (21.2)	4.5 (4.0 to 5.1)	3.6 (3.4 to 4.0)	1.41 (1.19 to 1.67)
	40 to 49.9	140/852 (16.4)	3.2 (2.7 to 3.8)	153/612 (25.0)	5.4 (4.6 to 6.3)	4.1 (3.6 to 4.6)	1.67 (1.32 to 2.19)
	≥50	73/268 (27.2)	5.9 (4.7 to 7.4)	80/237 (33.8)	7.4 (6.0 to 9.3)	6.6 (5.6 to 7.7)	1.26 (0.91 to 1.76)
LTF	Overall	1295/4414 (29.3)	6.1 (5.8 to 6.4)	616/2230 (27.6)	5.9 (5.5 to 6.4)	6.0 (5.8 to 6.3)	0.98 (0.89 to 1.08)
	18 to 24.9	148/349 (42.2)	9.9 (8.4 to 11.6)	18/42 (42.9)	8.6 (5.3 to 13.7)	9.7 (8.4 to 11.3)	0.87 (0.50 to 1.43)
	25 to 29.9	327/896 (36.5)	8.1 (7.2 to 9.0)	78/224 (34.8)	7.7 (6.2 to 9.7)	8.0 (7.2 to 8.8)	0.96 (0.74 to 1.23)
	30 to 39.9	528/2049 (25.8)	5.2 (4.8 to 5.7)	304/1115 (27.3)	5.8 (5.2 to 6.5)	5.4 (5.0 to 5.8)	1.12 (0.97 to 1.29)
	40 to 49.9	223/852 (26.2)	5.1 (4.5 to 5.8)	165/612 (27.0)	5.8 (5.0 to 6.7)	5.4 (4.9 to 5.9)	1.13 (0.92 to 1.39)
	≥50	69/268 (25.8)	5.6 (4.4 to 7.0)	51/237 (21.5)	4.7 (3.6 to 6.2)	5.2 (4.3 to 6.2)	0.85 (0.58 to 1.24)
Retention‐in‐care	Overall	1151/4414 (26.1)	5.4 (5.1 to 5.7)	543/2230 (24.3)	5.2 (4.8 to 5.7)	5.3 (5.1 to 5.6)	0.97 (0.87 to 1.07)
	18 to 24.9	69/349 (19.8)	4.6 (3.6 to 5.8)	10/42 (23.8)	4.8 (2.6 to 8.9)	4.6 (3.7 to 5.8)	1.04 (0.48 to 2.03)
	25 to 29.9	209/896 (23.3)	5.1 (4.5 to 5.9)	49/224 (21.9)	4.9 (3.7 to 6.4)	5.1 (4.5 to 5.8)	0.94 (0.68 to 1.29)
	30 to 39.9	557/2049 (27.2)	5.5 (5.0 to 6.0)	298/1115 (26.7)	5.7 (5.1 to 6.4)	5.6 (5.2 to 5.9)	1.04 (0.90 to 1.20)
	40 to 49.9	251/852 (29.5)	5.8 (5.1 to 6.5)	139/612 (22.7)	4.9 (4.1 to 5.8)	5.4 (4.9 to 6.0)	0.84 (0.68 to 1.05)
	≥50	65/268 (24.3)	5.2 (4.1 to 6.9)	47/237 (19.8)	4.4 (3.3 to 5.8)	4.8 (4.0 to 5.8)	0.83 (0.56 to 1.23)

a Exposed = male, unexposed = female.

### 3.2 Predictors of attrition at ten years on ART

**Figure 2:**
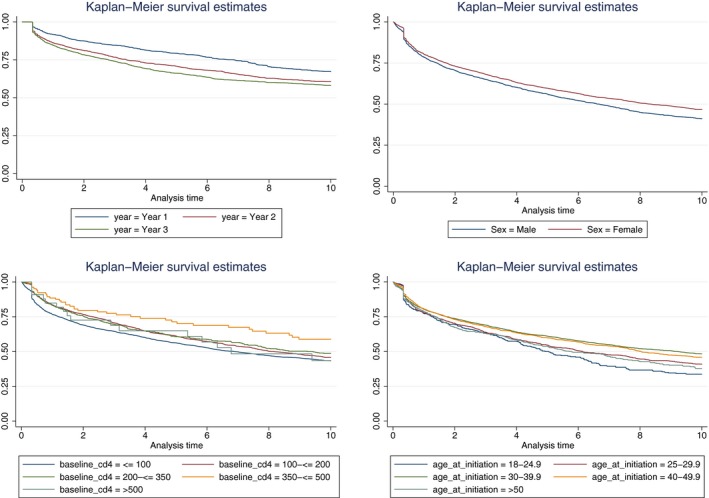
Crude Kaplan–Meier survival curves for attrition by ten years stratified by age at ART initiation, year of ART initiation, sex and baseline CD4 count. Log‐rank *p* < 0.001 for all. ART, antiretroviral treatment.

Cox proportional hazards models looking at the predictors of ten‐year attrition demonstrate several baseline factors that affect long‐term outcomes and that these can differ by sex (Table 3). In adjusted models among both females and males, those who initiated ART in the second and third years of South Africa's ART programme were more at risk of experiencing attrition over ten years than those who initiated in the first year of the programme; however, the risk was slightly lower for males than females (females in Y2: adjusted HR (aHR): 1.26, 95% CI: 1.11 to 1.43; females in Y3: aHR: 1.39, 95% CI: 1.24 to 1.57; males in Y2: 1.17, 95% CI: 0.98 to 1.38; males in Y3: 1.23, 95% CI: 1.05 to 1.45). Being South African (female: aHR: 1.57, 95% CI: 1.17 to 2.09; male: 1.77, 95% CI: 1.26 to 2.47) and having anaemia (female moderate: aHR: 1.32, 95% CI: 1.17 to 1.48; female severe: 1.83, 95% CI: 1.51 to 2.22; male moderate: aHR: 1.31, 95% CI: 1.11 to 1.55; male severe: aHR: 2.06, 95% CI: 1.57 to 2.69) and being underweight (female aHR: 1.33, 95% CI: 1.17 to 1.51; male aHR: 1.15, 95% CI: 1.00 to 1.32) were predictive of an increased risk of ten‐year attrition for both males and females, while initiating ART on AZT (female aHR: 1.37, 95% CI: 1.01 to 1.87) or on a protease inhibitor (female aHR: 1.30, 95% CI: 1.11 to 1.51) were predictive of increased risk for females only. In comparison, being older (30 to 30.9 years: aHR: 0.72, 95% CI: 0.61 to 0.85; 40 to 49.9: aHR: 0.74, 95% CI: 0.61 to 0.89) was protective against 10‐year attrition for females while, for men, having TB at ART initiation was protective (aHR: 0.76, 95% CI: 0.64 to 0.90). Crude Kaplan–Meier ten‐year survival estimates of attrition by age at ART initiation, year of ART initiation, sex and baseline CD4 count indicate that differences in attrition persist with these baseline stratifications (Figure 2).

**Table 3:** Predictors of attrition at ten years of ART among patients who initiated ART between April 2004 and March 2007^a^

	Female (N = 4414)			Male (N = 2230)		
	n/N (%)	Crude HR (95% CI)	Adjusted HR (95% CI)	n/N (%)	Crude HR (95% CI)	Adjusted HR (95% CI)
ART initiation cohort						
Year 1	514/1262 (40.7)	1.00	1.00	264/562 (47.0)	1.00	1.00
Year 2	691/1471 (47.0)	1.30 (1.16 to 1.46)	1.26 (1.11 to 1.43)	382/718 (53.2)	1.20 (1.03 to 1.40)	1.17 (0.98 to 1.38)
Year 3	802/1681 (47.7)	1.40 (1.24 to 1.55)	1.39 (1.24 to 1.57)	485/950 (51.1)	1.20 (1.03 to 1.40)	1.23 (1.05 to 1.45)
Nationality						
Non‐South African	55/162 (34.0)	1.00	1.00	42/117 (35.9)	1.00	1.00
South African	1951/4250 (45.9)	1.55 (1.18 to 2.02)	1.57 (1.17 to 2.09)	1089/2113 (51.5)	1.76 (1.29 to 2.40)	1.77 (1.26 to 2.47)
Missing	1/2 (50.0)					
Age at ART initiation (years)						
1 8 to 24.9	198/349 (56.7)	1.00	1.00	26/42 (61.9)	1.00	1.00
25 to 29.9	451/896 (50.3)	0.86 (0.73 to 1.02)	0.90 (0.75 to 1.08)	116/224 (51.8)	0.90 (0.59 to 1.37)	0.96 (0.60 to 1.52)
30 to 39.9	853/2049 (41.6)	0.67 (0.57 to 0.78)	0.72 (0.61 to 0.85)	540/1115 (48.4)	0.83 (0.56 to 1.24)	0.88 (0.58 to 1.36)
40 to 49.9	363/852 (42.6)	0.67 (0.56 to 0.79)	0.74 (0.61 to 0.89)	318/612 (52.0)	0.89 (0.60 to 1.33)	0.96 (0.62 to 1.48)
>50	142/268 (53.0)	0.90 (0.73 to 1.12)	1.07 (0.85 to 1.35)	131/237 (55.3)	0.96 (0.63 to 1.46)	1.08 (0.69 to 1.71)
CD4 count at ART initiation (cells/mm^3^)						
0 to 100	1052/2229 (47.2)	1.00	1.00	714/1355 (52.7)	1.00	1.00
101 to 200	685/1569 (43.7)	0.87 (0.79 to 0.95)	0.90 (0.81 to 1.01)	218/661 (48.1)	0.87 (0.76 to 0.99)	0.95 (0.83 to 1.10)
201 to 350	212/491 (43.2)	0.85 (0.74 to 0.99)	0.88 (0.74 to 1.04)	79/175 (44.9)	0.81 (0.64 to 1.02)	0.83 (0.64 to 1.07)
351 to 500	24/77 (31.2)	0.52 (0.35 to 0.78)	0.53 (0.34 to 0.83)	13/27 (48.2)	0.89 (0.51 to 1.54)	1.20 (0.65 to 2.20)
>500	11/24 (45.8)	0.83 (0.46 to 1.51)	0.96 (0.51 to 1.82)	5/9 (55.6)	1.23 (0.51 to 2.95)	1.30 (0.54 to 3.16)
Missing	23/24 (96.0)			2/2 (100.0)		
WHO Stage at ART initiation						
Stage 1/2	1104/2486 (44.4)	1.00	1.00	496/1014 (48.9)	1.00	1.00
Stage 3/4	903/1928 (46.8)	1.10 (1.00 to 1.20)	0.94 (0.84 to 1.06)	635/1216 (52.2)	1.11 (0.99 to 1.25)	1.09 (0.94 to 1.26)
Anaemia at ART initiation^b^						
None	594/1513 (39.3)	1.00	1.00	397/880 (45.1)	1.00	1.00
Mild	335/839 (39.9)	1.01 (0.88 to 1.16)	1.02 (0.88 to 1.17)	328/656 (50.0)	1.17 (1.01 to 1.36)	1.12 (0.96 to 1.31)
Moderate	829/1682 (49.3)	1.40 (1.26 to 1.55)	1.32 (1.17 to 1.48)	320/576 (55.6)	1.40 (1.21 to 1.63)	1.31 (1.11 to 1.55)
Severe	182/307 (59.3)	1.94 (1.64 to 2.29)	1.83 (1.51 to 2.22)	80/112 (71.4)	2.19 (1.72 to 2.78)	2.06 (1.57 to 2.69)
Missing	67/73 (91.8)			6/6 (100.0)		
BMI at ART initiation^c^						
Underweight	347/645 (53.8)	1.44 (1.27 to 1.63)	1.33 (1.17 to 1.51)	313/556 (56.3)	1.28 (1.12 to 1.47)	1.15 (1.00 to 1.32)
Normal	903/2101 (43.0)	1.00	1.00	634/1322 (48.0)	1.00	1.00
Overweight	369/895 (41.2)	0.97 (0.86 to 1.10)	0.95 (0.83 to 1.08)	73/176 (41.5)	0.81 (0.64 to 1.04)	0.86 (0.67 to 1.10)
Obese	191/444 (43.0)	1.02 (0.87 to 1.19)	1.05 (0.89 to 1.25)	13/29 (44.8)	0.82 (0.47 to 1.42)	0.80 (0.46 to 1.39)
Missing	197/329 (60.0)			98/147 (66.7)		
TB at ART initiation						
No	1680/3741 (44.9)	1.00	1.00	888/1726 (51.5)	1.00	1.00
Yes	327/673 (48.6)	1.11 (0.99 to 1.25)	0.99 (0.86 to 1.15)	243/504 (48.2)	0.88 (0.76 to 1.01)	0.76 (0.64 to 0.90)
Baseline ART regimen						
NRTI						
Stavudine	1952/4308 (45.3)	1.00	1.00	1088/2152 (50.6)	1.00	1.00
Zidovudine	55/106 (51.9)	1.33 (1.02 to 1.74)	1.37 (1.01 to 1.87)	43/78 (55.1)	1.21 (0.89 to 1.65)	1.28 (0.92 to 1.78)
PI						
No	1699/3844 (44.2)	1.00	1.00	1126/2209 (51.0)	1.00	1.00
Yes	308/570 (54.0)	1.36 (1.21 to 1.54)	1.30 (1.11 to 1.51)	5/21 (23.8)	0.36 (0.15 to 0.87)	0.34 (0.11 to 1.05)

CI, confidence interval; HR hazard ratio, PI, protease inhibitor.

Hazard ratio estimated from a Cox proportional hazard model.

a Attrition: loss to follow‐up and death.

b Anaemia is defined as: none if haemoglobin ≥12 for females and ≥13 for males; mild if haemoglobin 11.0 to 11.9 for females and 11.0 to 12.9 for males; moderate if haemoglobin between 8.0 and 10.9 for females and males; and severe if haemoglobin <8.0 for females and males (according to WHO guidelines).

c BMI categories are defined as: underweight if calculated BMI is <18.5 (kg/m^2^); normal if BMI is between 18.5 and 24.9 kg/m^2^; overweight if BMI is between 25.0 and 29.9 kg/m^2^ and obese if BMI is ≥30.0 kg/m^2^.

### 3.3 Patient perceptions and experiences with treatment

The mixed methods approach allowed us to understand the experiences of patients who remained in care for a full decade and gain insight into barriers and facilitators of long‐term retention. Of the 24 patients interviewed, 15 were female and a third were younger than 30 at the time of ART initiation, similar to the larger cohort (Table 4).

**Table 4:** Characteristics of the 24 patients interviewed who initiated ART between March 2004 and April 2005

	N (%)
Sex	
Male	9 (37.5)
Female	15 (62.5)
Age at time of interview (years)	
25 to 44	10 (41.7)
45 to 54	12 (50.0)
55+	2 (8.3)
Age at ART initiation (years)	
<30	8 (33.3)
30 or older	11 (45.8)
Missing	5 (20.8)
Has a spouse/partner	22 (91.7)
Has children	21 (87.5)
Employment	
Full‐time	7 (29.2)
Part‐time	6 (25.0)
Self‐employed	3 (12.5)
Unemployed	7 (29.2)
Missing	1 (4.2)
First CD4 count (cells/mm^3^)	
<50	7 (29.2)
51 to 100	2 (8.3)
101 to 150	1 (4.2)
151 to 200	7 (29.2)
>200	3 (12.5)
Missing/do not know	3 (12.5)
Pregnant at ART initiation	3 (12.5)
Had at least one drug reaction	21 (87.5)
Stopped treatment at least once	6 (25.0)
Disclosed HIV status to partner and/or family member	24 (100.0)
Disclosed to others outside family unit	
No	12 (50.0)
Yes	4 (16.7)
Missing	8 (33.3)

#### 3.3.1 Treatment initiation

When asked about their experiences initiating ART, participants reported that the clinic provided emotional and psychological support as treatment became available and that supportive counselling and informal support groups were crucial links to treatment initiation (Table 5). Many expressed gratitude at the arrival of ART, indicating that they had watched too many loved ones die of AIDS. Additionally, several patients identified the need to support family as a key reason for starting treatment. Prior to treatment initiation, the fear of death frequently impacted patient lives; once on ART this was alleviated, but some still experienced stigma and rejection when they disclosed their status, presenting an early threat to retention and adherence. However, all those interviewed had disclosed their HIV status to at least one family member, likely due to ART initiation guidelines stipulating this as a criterion during the early phase of treatment rollout; several indicated that this disclosure had helped them stay in care.

**Table 5:** Qualitative passages exploring patient experiences during the different stages of treatment

Stage	Barrier/facilitator	Theme	Descriptor	Example quote
Treatment initiation	Facilitator	Clinic provided emotional and psychological support as treatment became available	Female, Age 57	“You know, immediately when I started joining this group, I started accepting that here I am and I'm HIV positive and here are other people, and that sister was so brilliant. You know she even used to joke about HIV and we happened to laugh. Even we were sick, but we happened to laugh. She used to say you know what? The treatment is coming, and HIV is like a dog.”
			Female, Age 38	“Yo, when I'm coming to [the clinic], they welcome with open hand, you know that welcome. I was so happy getting counselling, true counselling, getting the treatment, no problems.”
	Facilitator	Supportive counselling and informal support groups were a crucial link to treatment initiation	Male, Age 45	“Yes, they give me a counselling. They give me a counselling. They also told me if I'm positive, but it's not the end of the world.”
			Female, Age 41	“In the support groups we were always speaking about these drugs. I didn't want class anymore I just wanted drugs because I was ready for drugs.”
	Facilitator	Many expressed gratitude at the arrival of ART, indicating that they had watched too many loved ones die of AIDS	Female, Age 54	“After my husband passed away, my daughter passed away, a lot of people in my community passed away because of HIV positive. So I think I'm lucky to have the treatment.”
			Female, Age 52	“I said to them I'm ready to take it [treatment]. I was so happy, because I lost the relatives, because of HIV. So I was so blessed, and feel really honoured.”
	Facilitator	Need to support and look after family as a key reason for initiating treatment	Female, 54	“It was not an easy job, but because I want to live for my children, I, I thought it's a good idea”
			Female, Age 57	“We vow to ourselves, we said no we don't want to leave our children no, no, I'm not going to die, I will wait for that, for that treatment…Some were vomiting… but we kept on saying no, we don't want to die.”
	Barrier	Stigma and rejection when they disclosed their status	Female, Age 57	“So I have to move from his shack because I could see now that he doesn't even want to see me anymore… it was like I came with a bulk of HIV.”
	Barrier	Stigma and rejection when they disclosed their status	Female, Age 57	“So I have to move from his shack because I could see now that he doesn't even want to see me anymore… it was like I came with a bulk of HIV.”
Retention and adherence	Facilitator	Understanding what was at stake along with a determination to stay healthy were key motivators to continued treatment and retention	Female, Age 45	“One thing that motivates me…If I didn't have my treatment I think I was supposed to be passed away a long time ago. I even have two kids after I was diagnosed…two beautiful daughters.”
			Male, Age 54	“My future is bright. You know uh, if I may? I'm not saying it will happen, if I may die, I won't die with the cause of AIDS. Do you know that? I might die with something else? But not with this.”
	Facilitator	A strong support network helped individuals overcome the negativity associated with being HIV positive	Male, Age 45	“Yes, they [family] gave me support. If I'm short with something, they give me that thing.”
			Female, Age 45	“But to me you know it was emotional at the same time again and now my mother said to me give me, I still remember that day, I never forget that day he gave me a hug. I think after some years she never gave me a hug, but that day my mother gave me a hug. And said don't worry, we'll go through together.”
	Facilitator	Improved quality of life experienced by individuals feeling healthy and happy on treatment gave them hope for the future	Male, Age 54	“My future is bright. …if I may die, I won't die with the cause of AIDS. Do you know that? I might die with something else? But not with this.”
	Facilitator	Empathetic and supportive clinic staff who encouraged patients to adhere to their treatment	Male, Age 45	“The way they have treated us, they treat me nicely. It's the thing that keeps me coming.”
			Female, Age 45	“He said to me you know what I want to give you the confidence that, just because today you are HIV you're not going to die. I want you to live. You're not going to die.”
	Barrier	Side effects after initiation ranged from mild to moderate symptoms including vomiting, weakness, dizziness, skin rashes, diarrhoea and headaches, to long‐term symptoms such as respiratory issues, weight gain, sexual dysfunction and extremity pain	Female, Age 45	“The only thing that I had after they changed me from that efavirenz and Kaletra my feet…my feet were painful. But then I came to the doctor and then I tell the doctor that ai no my feet are paining and then they gave me the tablets for that.”
	Barrier	Travel was a reason patients had on occasions missed doses or stopped treatment	Male, Age 49	“I went away [for work] for a month, and then where I was, there's my treatment finished, and I couldn't come back to the clinic.”
			Male, Age 45	“I was at a funeral at home, I forget my tablets, my treatment here. So I went there in Eastern Cape without repeat, that's the only time I forget to take treatment, yes.”
	Barrier	Stigma and fear of being seen taking medication	Female, Age 45	“You know my treatment it was very challenging. Because when you go to a place like when you are working you don't have time to drink it in the specific time. Sometimes you forgot oh I must take it.”
			Female, Age 45	“I don't want to lie to you. Ja, they gave us time, but that time I'm telling you, because at 7 o'clock I was on the street, you understand. Going to work. I can't take my tablets in my bag and my medication and my bottle. Because now I'd rather this thing we afraid of people. What tablet is this? You understand. And taking them with your container, and on that container it was written because even the TV they were taking them out. So it was very scary.”
	Barrier	Hospitalization	Female, Age 54	“I had to leave it for three months and then they started the new treatment again that's when I started to get better.”
	Barrier	Deterioration in attitudes of some providers as a result of staff shortages and increased patient volumes	Female, Age 57	“And the staff were kind of busy, and sometimes they get violent. They get angry because people were many by that time…They were always shouting and cross, angry…”
	Barrier	Long queues sometimes meant patients had to return to the clinic multiple times to collect medication	Male, Age 49	“Sometimes you come here, you don't get medication, you have to come back tomorrow because it's late…they couldn't finish…”

#### 3.3.2 Retention and adherence

When asked about their experiences on ART, staying in care and adhering to medication, even though participants were grateful to have access to treatment, several factors still negatively influenced early adherence. Side effects after initiation were mentioned by 63% (n = 15) of participants, ranging from mild to moderate symptoms including vomiting, weakness, dizziness, skin rashes, diarrhoea and headaches, to long‐term symptoms such as respiratory issues, weight gain, sexual dysfunction and extremity pain. In addition, travel, hospitalization, abuse, stigma and fear of being seen taking medication were cited as barriers to continued treatment and, for some, led to temporary missed doses or stopped treatment. Among the twenty‐four interviewed, four reported a gap in treatment ranging between two weeks and six months. Health system challenges also posed a threat to retention and adherence. Deterioration in attitudes of some providers as a result of staff shortages and increased patient volumes affected the emotional and psychological wellbeing of many patients and threatened their retention‐in‐care. Furthermore, long queues sometimes meant patients had to return to the clinic multiple times to collect medication which was a barrier to continued care.

Despite these challenges, even when patients did describe gaps in treatment, they reported returning to care as quickly as they were able. Understanding what was at stake along with a determination to stay healthy were key motivators to continued treatment and retention. A strong support network helped individuals overcome the negativity associated with being HIV positive, and the improved quality of life experienced by individuals on treatment gave them hope for the future and encouraged continued treatment.

## 4 Discussion

In this mixed methods analysis of 6644 adults who initiated ART between April 2004 and March 2007, among those who did not transfer their treatment to another clinic, 79.5% and 35.1% were retained, alive and in care at the same facility at twelve months and ten years respectively. This 12‐month retention rate is comparable to the 77% summarized retention in a meta‐analysis using 2008 to 2013 LMIC data 38. Few sub‐Saharan African studies have reported data on long‐term ART outcomes – two from Nigeria (ten‐ and seven‐year follow‐up), one from Uganda (ten‐year follow‐up), one from Zimbabwe (ten‐year follow‐up), one from Mozambique (nine‐year follow‐up) and one from South Africa (twelve‐year follow‐up) 23, 24, 25, 26, 27, 28. These studies show mixed results, with retention ranging from 37% to 83%, mortality from 1% to 24% and LTF from 5% to 51%; our observed ten‐year 18.5% mortality and 28.8% LTF from the initiating clinic lie within these ranges when formal transfers are included. When those who transferred out of the facility are excluded, ten‐year mortality is slightly higher at 25.4% and LTF, although still in range compared to other studies, is also higher at 39.6%. Lower rates of mortality and LTF seen in some studies may be the result of intensive monitoring and enhanced patient retention interventions, including counselling, appointment reminders and active tracing 25. Additionally, linking our mortality data to the VRS would have resulted in higher mortality than observed in patient files, as demonstrated by previous work done by our group that found that 37% of those lost to follow‐up had actually died 36. Although the overall ten‐year retention of 25% may seem low, over a quarter of those no longer in care at the clinic after ten years are known to have transferred to another clinic for treatment, aligning with increasing treatment availability and known efforts to down‐refer stable patients to primary health clinics (PHCs). As such, this low retention does not necessarily signify poor ten‐year retention overall but more likely indicates that patients have continued treatment elsewhere. Furthermore, linking with the VRS allows us to conclude that at least some of the LTF observed at this clinic is the result of patients silently transferring care to other facilities rather than being lost to care completely as these patients do not appear in the Registry 39; however, not all patients who were lost to follow‐up, but not in the VRS can be assumed to be in care, especially those who were lost in the later years of the study and may be alive, but not on treatment.

This large patient cohort provides valuable insight into the long‐term ART outcomes in South Africa. Twelve‐month treatment outcomes were generally encouraging despite early health systems barriers and drug toxicity reported by patients, as well as advanced disease at initiation. Now, with earlier disease‐stage treatment initiation, less toxic drug regimens and better patient retention interventions, we would hope to see improved short‐term outcomes among future cohorts 26. However, evidence that high rates of LTF continue over ten years is of concern despite the likelihood that many of these patients may have received care elsewhere. Our qualitative data emphasize the important role supportive clinic staff played prior to, and during, the rollout of ART along with social support were also critical aspects of adherence and retention, with the lack of a supportive environment resulting in significant barriers 40, 41, 42, 43, 44. These results suggest that over time clinic barriers (such as long waiting times and staff attitude) may pose a threat to patient retention, factors that must be considered as we think about ways to support patients as they embark on lifelong treatment.

As the South African ART programme has matured, even though patients still present later than hoped, they are generally initiating ART healthier than they were during the early years of ART availability 17. Although our qualitative data do not address this directly, it is possible that, in this more recent context, the effects of treatment may be less dramatic for individual patients and the negative impact of ART drug side effects may outweigh the perceived benefits of treatment, possibly leading to a decrease in adherence and retention 45. This is an important idea to consider as the universal “treat all” approach is implemented in South Africa. Additionally, the strong feelings of gratitude for receiving ART, along with witnessing the deaths of loved ones and community members, were strong retention and adherence facilitators for these early initiators; these historical facilitators may not be as effective in the current treatment era, highlighting the need for research into, and interventions towards, decreasing barriers to retention. Furthermore, given the strong evidence that clinic support is a key facilitator of adherence and retention, interventions such as adherence clubs and support groups will likely help improve patient self‐efficacy and adherence 46, 47, 48. Additionally, efforts to decrease clinic barriers and decongest facilities by decanting stable patients to repeat prescription collection strategies that minimize the time required to collect medication and allow collection of more months of medication are also likely to have a positive impact on long‐term retention and adherence. Evidence is emerging on the effectiveness of some of these support interventions on adherence and long‐term retention 26, 49.

This study has important limitations. Firstly, our quantitative analysis of patients initiating in the first years of the treatment programme may have limited generalizability to current programming as the treatment initiation context has changed substantially. Secondly, in our qualitative analysis, we asked participants to remember details from ten years ago, including treatment gaps, possibly resulting in recall bias. Thirdly, we only spoke to people who had successfully navigated treatment at one facility for over ten years – the ART programme's “success stories.” This excludes the perspectives and experiences of patients who transferred to another clinic or dropped out of care. Lastly, the study clinic is a gold standard facility that started treating patients earlier than many of the surrounding PHCs. As such, despite patients being able to be down‐referred to other treatment facilities over time, some have chosen to continue care at the study clinic, raising the question of whether these patients are somehow exceptional patients rather than the average patient. Despite these limitations, this study offers invaluable insight into patient outcomes and experiences over a longer period of time than has previously been reported. As other resource‐limited countries navigate the transition to “treatment for all” and adjust to a changing HIV treatment landscape, this study may be relevant in providing evidence of long‐term treatment benefits as well as lessons from those who have successfully been on treatment over a long period of time.

## 5 Conclusions

South Africa's expansion of ART to over 3.4 million people since programme inception has been commendable. Our findings are important as this is one of the first studies from South Africa that demonstrates long‐term programme successes with declining mortality, along with inspiring examples of patients who have successfully been on treatment for over ten years. However, it also highlights substantial barriers to long‐term retention which may be particularly relevant as South Africa navigates the transition to “treatment for all.” As more people become eligible for ART and initiate at earlier stages of disease, appropriate interventions and support are needed to ensure long‐term programme success continues.

## Competing interest

The authors declare that they have no competing interests.

## Authors’ contribution

CH and MPF conceptualized the study. CH, MM and SP designed the study. CH, AM and SP conducted the analysis. CH, AM, ANH and SP prepared the original draft. CH, AM, SF, ANH, MM, LL and MPF reviewed and edited the draft.

## Supporting information


**Table S1.** Coding framework for the qualitative analysis.
