# Supplementary material for: “My future is bright…I won't die with the cause of AIDS”: ten‐year patient ART outcomes and experiences in South Africa
Source: J Int AIDS Soc. 2018 Oct 14;21(10):e25184. doi: 10.1002/jia2.25184 (PMC6186968; doi:10.1002/jia2.25184)
Supplement: Supplementary file 1 — Table S1. Coding framework for the qualitative analysis. [file JIA2-21-e25184-s001.docx]

#### **Supplementary Table 1: Coding framework for the qualitative analysis**

| **Category** | **Theme** | **Code** | **Sub-code** |
| --- | --- | --- | --- |
| **Experiences Pre-Testing** | Decision to test | Health factors | Worsening of physical symptoms |
|  |  |  | Pregnancy |
|  |  | Intrinsic factors | Desire to survive |
|  |  |  | Acceptance of possible HIV positive status |
|  |  |  | Lack of understanding of how HIV positive |
|  |  |  | Denial |
|  |  |  | Fear of dying |
|  |  | External factors | Non-disclosure by partner of HIV status |
|  |  |  | Rape |
|  |  |  | Knowledge that treatment not yet available |
|  |  |  | Illness of others (child/spouse) |
|  |  |  | Forced testing at ANC |
|  |  | Support networks | Partner |
|  |  |  | Family |
|  |  |  | Private doctor |
|  | Personal experiences | Beliefs |  |
|  |  | Fears |  |
|  |  | Physical health |  |
|  |  | Quality of Life |  |
|  |  | Family Support |  |
|  |  | Marital issues |  |
|  |  | Faith and religion |  |
|  | Experiences at clinic | Pre Test Counselling |  |
|  |  | Support from clinic staff |  |
| **Experiences Post-Test and Pre-Treatment** | Decision to initiate treatment | Health factors | Waiting for CD4 count to reach eligibility threshold |
|  |  |  | Worsening of physical symptoms |
|  |  | Intrinsic factors | Gratitude to be among first to be offered ART |
|  |  |  | Desire to survive |
|  |  |  | Fear of perceived side effects |
|  |  | External factors | Rape |
|  |  |  | Death of others prior to treatment availability |
|  |  |  | Treatment becoming available |
|  |  | Support networks | Family/partner |
|  |  |  | Support groups |
|  |  |  | Clinic staff |
|  |  |  | Other HIV-positive patients waiting for ART |
|  | Personal experiences | Beliefs |  |
|  |  | Fears |  |
|  |  | Physical health |  |
|  |  | Quality of Life |  |
|  |  | Family Support |  |
|  |  | Marital issues |  |
|  |  | Faith and religion |  |
|  | Experiences at clinic | Counselling |  |
|  |  | Support from clinic staff |  |
|  |  | Support groups |  |
|  | Barriers | Clinic Factors | Eligibility requirements |
|  |  |  | Treatment availability |
|  |  | Individual Factors |  |
|  |  | Other |  |
|  | Facilitators | Clinic Factors | Supportive clinic staff |
|  |  | Individual Factors | Physical health |
|  |  | Other |  |
| **Experiences on Treatment** | Decision to continue care and adhere to ART | Health factors | Side effects |
|  |  |  | Physical wellbeing |
|  |  | Intrinsic factors | Grateful to be on treatment |
|  |  |  | Recognition of drugs role in quality of life improvement |
|  |  |  | Spiritual faith |
|  |  |  | Belief in own survival |
|  |  | External factors | Being a support to others |
|  |  |  | Life-style changes |
|  |  |  | Being a parent/having children to support |
|  |  |  | Stigma |
|  |  |  | Strict drug timelines |
|  |  |  | Work/employment |
|  |  |  | Availability of treatment |
|  |  | Clinic environment | Poor staff attitudes |
|  |  |  | Long queues |
|  |  | Support networks | Family |
|  |  |  | Partners/spouses |
|  |  |  | Employers |
|  |  |  | Clinic staff |
|  | Personal experiences | Beliefs |  |
|  |  | Fears |  |
|  |  | Lifestyle | Alcohol |
|  |  |  | Smoking |
|  |  | Family support |  |
|  |  | Marital issues |  |
|  |  | Quality of life |  |
|  |  | Support |  |
|  |  | Faith and religion |  |
|  |  | Physical health | Side effects |
|  |  |  | Improvement of health on ART |
|  |  | Work life experiences | Stigma |
|  |  |  | Support |
|  |  |  | Privacy to take medication |
|  |  |  | Travel |
|  | Experiences at clinic | Availability of ART |  |
|  |  | Long queues |  |
|  |  | Staff attitudes |  |
|  | Barriers | Clinic Factors | Long queues |
|  |  |  | Unsupportive staff |
|  |  | Individual Factors | Side effects |
|  | Facilitators | Clinic Factors | Supportive clinic staff |
|  |  | Individual Factors |  |
| **Cross-cutting themes** | Attitudes Towards Future | Positive |  |
|  |  | Fears |  |
|  |  | Challenges |  |
|  |  | Negative |  |
|  | Disclosure/ Non-disclosure | Disclosure | Workplace |
|  |  |  | Community |
|  |  |  | Family |
|  |  |  | Friends |
|  |  |  | Partner |
|  |  | Non-Disclosure | Family |
|  |  |  | Outside Family Unit |
|  | Stigma | Experiences of stigma from others towards self | Community |
|  |  |  | Family |
|  |  |  | Workplace |
|  |  | Changes over time |  |
|  | Family Context Over Time | Acceptance |  |
|  |  | Challenges |  |
|  |  | Children |  |
|  |  | Support |  |
|  | Quality of Care | Acceptable |  |
|  |  | Accessible |  |
|  |  | Care Navigation |  |
|  |  | Effective |  |
|  |  | Efficient |  |
|  |  | Equitable |  |
|  |  | Patient Satisfaction |  |
|  |  | Safe |  |
